# Evidence according to Cochrane Systematic Reviews on Alterable Risk Factors for Anastomotic Leakage in Colorectal Surgery

**DOI:** 10.1155/2020/9057963

**Published:** 2020-01-03

**Authors:** Bradley Wallace, Fabia Schuepbach, Stefan Gaukel, Ahmed I. Marwan, Ralph F. Staerkle, Raphael N. Vuille-dit-Bille

**Affiliations:** ^1^Department of Pediatric Surgery, Children's Hospital Colorado, USA; ^2^University of Zürich, Switzerland; ^3^Department of Orthopaedics and Traumatology, Cantonal Hospital Winterthur, Switzerland; ^4^Clarunis, Department of Visceral Surgery, University Centre for Gastrointestinal and Liver Diseases, St. Clara Hospital and University Hospital Basel, Switzerland; ^5^Department of Pediatric Surgery, University Children's Hospital of Basel, Switzerland

## Abstract

Anastomotic leakage reflects a major problem in visceral surgery, leading to increased morbidity, mortality, and costs. This review is aimed at evaluating and summarizing risk factors for colorectal anastomotic leakage. A generalized discussion first introduces risk factors beginning with nonalterable factors. Focus is then brought to alterable impact factors on colorectal anastomoses, utilizing Cochrane systematic reviews assessed via systemic literature search of the Cochrane Central Register of Controlled Trials and Medline until May 2019. Seventeen meta-anaylses covering 20 factors were identified. Thereof, 7 factors were preoperative, 10 intraoperative, and 3 postoperative. Three factors significantly reduced the incidence of anastomotic leaks: high (versus low) surgeon's operative volume (RR = 0.68), stapled (versus handsewn) ileocolic anastomosis (RR = 0.41), and a diverting ostomy in anterior resection for rectal carcinoma (RR = 0.32). Discussion of all alterable factors is made in the setting of the pre-, intra-, and postoperative influencers, with the only significant preoperative risk modifier being a high colorectal volume surgeon and the only significant intraoperative factors being utilizing staples in ileocolic anastomoses and a diverting ostomy in rectal anastomoses. There were no measured postoperative alterable factors affecting anastomotic integrity.

## 1. Introduction

While writings of Hippocrates and Celsus contain the first known references to intestinal suturing, the 19^th^ century advents of anesthesia and aseptic technique have permitted the evolution of modern visceral surgery [1]. Historically, Sir Astley Cooper is often credited with the first successfully sutured intestinal anastomosis in 1806. Travers then published further scientific inquiry of intestinal repair, and these techniques were further adapted in 1826 when Lembert introduced an inverted extramucosal suturing method to prevent invagination and ensure serosa to serosa repair [1]. In 1834 and 1841, respectively, Dupuytren and Appolito developed continuous multilayered suturing techniques [2]. Though technique adaptation has undergone minor developments since, most recent changes have been to anastamotic materials, initially with the creation of newer sutures and, most recently, creation of the circular stapling devices, invented in Russia in the 1960s and first described by Ravitch and Steichen in 1979 [3].

Presently, either single-stitch or continuous handsewn absorbable sutures or stapler devices with nonabsorbable staples are most commonly used [2]. Principally, the stapler device places two or more rows of staples and divides the tissue in between the staple lines [4, 5]. There are circular (EEA type) and linear (GIA type) stapling devices, the latter used for side-to-side anastomoses and the former used for end-to-end or end-to-side anastomoses [6]. To generate a viable anastomosis, factors like adequate perfusion, freedom from tension at the anastomotic site, and absence of distal obstruction and mesenteric twisting are favorable [7]. To bolster the anastomosis, on occasion omentoplasty (i.e., covering of the anastomosis with the greater omentum) may be performed [8].

Despite substantial progress in surgical techniques and imaging methods, anastomotic leakage remains a major complication following bowel surgery and carries a high rate of morbidity and mortality [9]. Reported colorectal leakage rates range between 4 and 26% and lead to increased hospital costs, lengths of stay, readmissions, reoperations, procedures, complications, and death [8, 10–13]. The International Study Group of Rectal Cancer (ISGRC) proposed defining anastomotic leakage (following anterior rectal resection) as “a defect of the integrity of the intestinal wall at the anastomotic site (including leakage originating from suture and staple lines of neorectal reservoirs) leading to a communication of the intra- and extraluminal compartments” [12]. Furthermore, the ISGRC recommends considering a pelvic abscess adjacent to the anastomosis as an anastomotic failure as well [12]. The escape of feces into the abdominal cavity may cause fever, fecal and/or sanguineous discharge from drains, abscess formation, septicemia, metabolic disturbance, and/or multiple-organ failure [10]. In hemodynamically stable patients, the first-line imaging modality to detect colorectal anastomotic leakage constitutes a triple (i.e., oral-, rectal-, and intravenous-) contrast abdominal and pelvic computed tomography (CT) scan, which can subsequently guide management ranging from CT-guided percutaneous abscess drainage to further surgery. Of note, sensitivity of CT imaging following colorectal anastomosis can vary between 60 and 100%; thus, a negative CT scan does not exclude anastomotic leakage [14, 15]. A clinical deterioration of the patient is thus the main indicator of anastomotic failure. Whereas typically on the fifth postoperative day patients suffering from anastomotic leakage will present with fever, elevated white blood cell counts, elevated C-reactive protein levels, abdominal discomfort, and intestinal paralysis, notably, anastomotic dehiscence may instead be clinically silent, especially in the setting of a diverting ostomy [8, 12, 16]. C-reactive protein levels are the most sensitive biochemical markers for a leak, with levels > 150 mg/l by postoperative day 3-5 worrisome [17]. In 2001, Bruce and colleagues suggested a grading system to categorize anastomotic leakages according to their clinical consequences: (i) *radiologic* (not leading to changes in treatment), (ii) *clinically minor* (causing prolonged hospital stay), and (iii) *clinically major* (necessitating intervention with a change in clinical management) [12]. Nevertheless, most authors and clinicians do not use a grading system in daily practice to categorize anastomotic leakage.

In order to reduce the anastomotic leakage rate, different pre-, intra-, and postoperative factors have been tested in the past. The aim of the present review was to briefly summarize the nonalterable factors of colorectal anastomotic leakage rates prior to evaluating alterable perioperative factors from the available Cochrane systematic reviews.

## 2. Methods

### 2.1. Literature Search

The Cochrane Central Register of Controlled Trials (The Cochrane Library Issue 5 of 12, May 2019 (http://onlinelibrary.wiley.com/cochranelibrary/search)) was searched using the following search terms: “*randomized AND* (*colonic OR colorectal OR rectal OR ileocolic OR intestinal*) *AND* (*anastomotic OR anastomosis OR anastomose*) *AND* (*failure OR leak OR leakage*),” revealing 733 hits (16 Cochrane reviews and 717 trials). Thereof, 13 Cochrane systematic reviews addressing 16 putative risk factors were identified.

For Medline/PubMed search, the following search term was used: “(colonic OR colorectal OR rectal OR ileocolic OR intestinal) AND (anastomotic OR anastomosis OR anastomose) AND (failure OR leak OR leakage).” Restricting to systematic reviews and meta-analyses, the Medline search resulted in 314 titles. The Medline search ended on the 25th of May 2019. Hereby, 11 Cochrane systematic reviews addressing 14 putative risk factors were identified.

**Figure 1:**
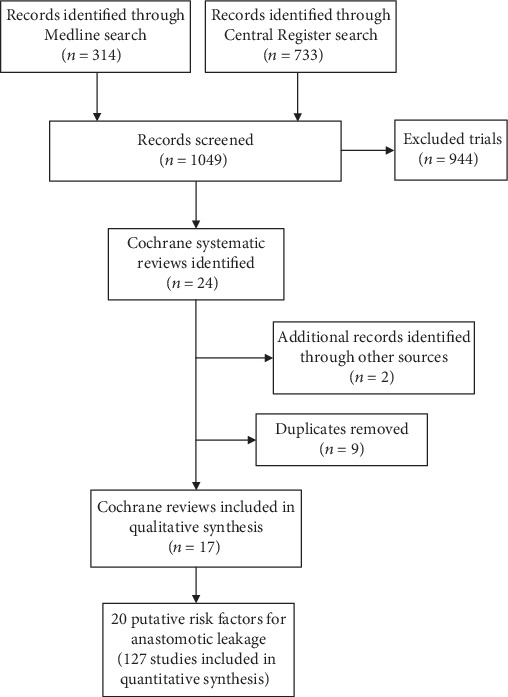
Study flow diagram.

In addition, two Cochrane systematic reviews (addressing two risk factors) were identified through unsystematic searches [4, 18] (Figure 1).

## 3. Results

Twenty alterable factors potentially affecting colorectal anastomotic leakage were assessed for pre-, intra-, and postoperative interventions. Evaluated factors for management included preoperatively mechanical bowel preparation versus no bowel preparation (I), mechanical bowel preparation versus rectal enema (II), preoperative chemoradiation versus radiation alone for stage II and III resectable rectal cancer (III), hospital volume (IV), surgeon's volume (V), surgeon's specialization (VI), and primary versus staged resection for obstruction from left-sided colorectal carcinoma (VII); included intraoperatively laparoscopic vs. open approach for rectal cancer (VIII) and sigmoid diverticulitis (IX), stapled vs. handsewn colorectal (X) and ileocolic (XI) anastomosis, omentoplasty (XII), single vs. double layer anastomosis (XIII), intraperitoneal agents for preventing adhesions (XIV), prophylactic anastamotic drainage (XV), covering ostomy (in anterior resection for rectal carcinoma) (XVI), and ileostomy or colostomy for left-sided colorectal anastomosis (XVII); and included postoperatively nasogastric decompression (XVIII), early enteral nutrition (XIX), and epidural versus opioids (XX) (Table 1). Among these, three factors significantly affected the incidence of anastomotic leakage: high (versus low) surgeon's operative volume (RR = 0.68), stapled (versus handsewn) ileocolic anastomosis (RR = 0.41), and a diverting ostomy in anterior resection for rectal carcinoma (RR = 0.32). The remaining 17 factors did not affect the incidence of anastomotic leakage.

## 4. Discussion

### 4.1. Grouping of Risk Factors for Colorectal Anastomotic Leakage

Putative risk factors may be grouped into alterable (i.e., laparoscopic vs. open surgery, postoperative feeding) and nonalterable (age, sex, height of anastomosis, etc.) risk factors. Furthermore, putative risk factors for colorectal anastomotic leakage may be grouped into surgical techniques (i.e., handsewn versus sutured anastomosis, single versus double layer suture) [4] and general risk factors [19]. We will begin our discussion of nonalterable factors prior to closer examination of the evidence regarding alterable perioperative factors.

### 4.2. Nonalterable Risk Factors for Colorectal Anastomotic Leakage

In the past, different nonalterable putative risk factors have been investigated, including sex [20, 21], age [22, 23], body mass index [20, 23], general morbidity of the patient/American Society of Anaesthesiologist Grade (ASA Grade) [13, 16, 24], malnutrition [13, 25], smoking [7, 13, 23, 26], elective versus emergency operation [16, 22–24], operative time [13, 20, 24], anemia [21, 27, 28], perioperative blood transfusion [13, 24], alcohol consumption [24, 29], renal disease [17], and height (i.e., distance to the dentate line) of the anastomosis [30]. As often these putative risk factors are nonalterable, they may not be assessed by randomized controlled trials. The highest level of evidence hence reflects meta-analyses including observational studies or observational studies with dramatic effects. Male sex, obesity, poor nutrition, high ASA score, advanced tumor stage, emergency surgery, smoking, comorbidities, renal disease, immune-suppressants, and history of radiotherapy are reported as preoperative nonalterable risk factors for anastomotic leakage [17]. Intraoperative risk factors consist of blood loss, necessity of blood transfusion, and duration of surgery longer than four hours [17]. Concerning the location of the anastomosis, the closer the colorectal anastomosis is to the anus, the higher the risk of leakage [30]; hence, extraperitoneal anastomoses show more complications than intraperitoneal colorectal anastomoses [4]. Other factors not assessed by RCTs are surgeon-related factors including surgeon's training [31], sleep deprivation [32], experience [33], and specialty [33].

### 4.3. Alterable Risk Factors for Colorectal Anastomotic Leakage Assessed by Cochrane Systematic Reviews

Given the increased morbidity, mortality, and hospital costs caused by colorectal anastomotic leaks [34], proper assessment of alterable risk factors must be made. This can be grouped into preoperative, intraoperative, and postoperative factors. Among putative risk factors, only some of them have been assessed by Cochrane systematic reviews.

### 4.4. Preoperative Risk Factors

#### 4.4.1. Mechanical Bowel Preparation

Mechanical bowel preparation to empty the colon from stool to prevent complications of infection and anastomotic leakage has been practiced dogmatically for over a century; however, recently, the accepted superiority of this practice has been called to question [35]. Previously touted as anastomotically protective [36], since the 1970s, numerous studies have compared the potential protection against the patient inconvenience as well as possible dangerous side effects of electrolyte imbalances, dehydration, and inflammation [37–39]. The French GRECCAR III multicenter trial was the first randomized control trial regarding rectal cancer surgery with and without mechanical bowel preparation which found no difference in anastomotic leakage and major morbidity rates between the two groups (though they did find higher risk of infection and overall morbidity in the no mechanical bowel prep group) [40]. Cochrane review from 13 RCTs over 4633 patients revealed that preoperative mechanical bowel cleaning of the colon had no difference on the primary outcome of colorectal anastomotic leakage nor on secondary outcomes of mortality, peritonitis, reoperation, wound infection, and infectious and noninfectious extra-abdominal complications. Therefore, mechanical bowel preparation should not be performed routinely but only in particular situations (such as if intraoperative colonoscopy will be performed) [41]. Furthermore, mechanical bowel preparation was compared to rectal enema by the same Cochrane review including 5 RCTs with 1210 patients, which also showed no difference in outcomes [41].

#### 4.4.2. Neoadjuvant Chemoradiation

Colorectal cancer is one of the most common malignant neoplasms in the Western World [42]. In Europe alone, more than 200,000 fatal incidences per year are reported [43]. A main pillar of therapy, surgical excision, is conducted whenever possible; however, studies show that excision alone is accompanied by local recurrence rates of 25% and carries a poor prognosis. It has been suggested that despite resection, the microscopic tumor remains in the suspensory apparatus of the colon [44], giving rise to local recurrence. Today, much lower recurrence rates can be achieved with total mesorectal excision (in rectal cancer) as well as with adjuvants of radiation and chemotherapy [45]. Although postoperative radiation therapy reduces local cancer recurrence as well [46], preoperative application has been shown to be superior as intact and well-oxygenated tissue allows for more adequate radiation doses and leads to higher tumor response [47]. Neoadjuvant chemoradiation can be used to both shrink and down-stage cancers, sometimes allowing for excision of prior unresectable tumors [48], making resection technically easier and increasing the rate of R0 resections [49]. Even in the setting of total mesorectal excision of rectal cancer, neoadjuvant radiation has been shown to further improve outcomes [50].

Cochrane review of 4 RCTs including 1151 patients analyzing the advantages of neoadjuvant chemoradiation compared to radiotherapy alone revealed a significantly lower rate of local rectal cancer recurrence after neoadjuvant chemoradiation. However, neoadjuvant chemoradiation corresponded with a higher incidence of acute toxicity. Assessing primary outcomes of disease-free survival along with secondary outcomes of overall survival and rectal anastomotic leakage rates revealed no difference between neoadjuvant radiation compared to chemoradiation therapy. There was no difference in the functional outcome (such as sphincter preservation) among the two groups [43].

#### 4.4.3. Hospital Volume, Surgeon's Specialty, and Surgeon's Experience

For many years, a higher volume concentration of care has been postulated to improve patients' outcomes in rare diseases [33]. In complex cancer surgeries by high-volume providers, better patient outcomes alongside improvements in training, research, and economic efficiency have led to service centralizations in many countries [51]. In several specializations (including colorectal surgery), a high-volume surgeon is believed to have greater experience that improves case selection as well as surgical technique and decision-making pre-, intra-, and postoperatively [52]. Furthermore, high-volume hospitals are believed to have an improved organization of care including a multidisciplinary teamwork approach and 24-hour availability of other specialties, as well as more research opportunities [53]. Cochrane review from Archampong and coworkers analyzed the effect of hospital volume, surgeon's specialty, and surgeon's experience on outcomes following colorectal surgery [33]. While including only nonrandomized and observational studies, 5-year survival was significantly improved for colorectal cancer patients treated by high-volume surgeons, in high-volume hospitals, or by colorectal specialists. Similarly, operative mortality was lower when high-volume or specialist surgeons were operating. Impact of hospital volume, surgeon's volume, and surgeon's specialty on the anastomotic failure rate following colorectal anastomosis was, respectively, tested by 8, 4, and 4 nonrandomized or observational studies, including more than 5000 patients for each comparison. Whereas hospital volume and surgeon's specialty had no effect on the colorectal anastomotic leakage rate, surgeon's volume was associated with a lower number of anastomotic leaks (relative risk 0.68) [33]. Nevertheless, quality of evidence is low, not only due to the design of included studies but also because of varying definitions of high-volume and colorectal specialists.

#### 4.4.4. Treatment of Obstructing Left-Sided Colorectal Cancer

Gastrointestinal neoplasms are a major cause of acute large bowel obstructions [54–57], for which, immediate bowel decompression is crucial [55]. Decompression may be achieved through resection (primary or staged), diversion, or stenting. In staged resection, initial diverting ostomy precedes secondary tumor resection with tertiary attempted closure of the ostomy. Primary resection is widely preferred for right-sided colonic malignancy [57–59]; however, a Cochrane review by De Salvo et al. attempted to compare primary versus staged resection for left-sided colorectal malignancy [18]. They identified one RCT that had to be excluded due to methodological weakness.

Stent insertion for acute mechanical bowel obstruction was first described by Dohmoto in 1991 [60]. Since then, self-expanding metal stent (SEMS) application has found widespread acceptance particularly in palliative situations [61]. SEMS has been shown to significantly reduce ICU admissions, need for ostomy creation, and lengths of hospital stay and has been shown to significantly improve quality of life for palliative patients [61, 62]. Alternatively, in acute obstructions, SEMS has been used as a bridge treatment to subsequent elective surgery in order to reduce complications caused by emergency surgery [54, 58, 62, 63]. Decreased complications and decreased hospital lengths of stay following SEMS insertion have been linked to cost savings in patients with operable cancer [54].

The success rate of intestinal SEMS insertion for large intestine tumor obstruction is usually over 90%, and consequent decompression occurs in most cases [63–66]. Major complications including bowel perforation leading to peritonitis (with theoretical malignant seeding) [66] and postinterventional death [63] occur rarely. However, alternate complications such as abdominal pain [64], stent migration [63, 65], mild bleeding, and tumor growth into the stent [64] occur more frequently (incidence of 13-42%) [64, 66]. There is no Cochrane systematic review comparing colonic stents versus emergency surgery for malignant colonic obstruction. Ribeiro et al. performed a meta-analysis of 4 RCTs including 125 patients and found that mortality, length of ICU stay, and early complications of both methods were similar, whereas SEMS had the advantage of lower risk of permanent stoma and earlier hospital discharge [67]. Anastomotic leakage was not addressed.

### 4.5. Intraoperative Risk Factors

#### 4.5.1. Laparoscopic vs. Open Surgery

Laparotomy, as an invasive procedure, is associated with considerable morbidity and long convalescence [68, 69]. In 1991, Jacobs et al. first described the feasibility of colectomy by video-assisted, endoscopic surgery, without laparotomy [70], which offered reduced postoperative morbidity, faster oral feeding, and shorter hospital stay [71]. Nowadays, laparoscopy is more prevalent and applied to both simple [72–74] and complex [75] surgical cases. Although technically more difficult, after a learning curve, laparoscopic resection has shown equivalent operative results to conventional open colectomy [76]. Cochrane review of 10 RCTs including 2505 patients comparing laparoscopic versus open total mesorectal excision for rectal cancer showed similar disease-free and overall survival, as well as similar tumor recurrences. Operative times were shorter with the open approach; however, the laparoscopic approach had decreased blood loss and shorter hospital stay. The incidence of colorectal/coloanal anastomotic leakage was not different between groups [77]. Cochrane review of 3 RCTS including 349 patients comparing laparoscopic versus open resection for sigmoid diverticulitis showed shorter operative time for the open approach, whereas postoperative pain was decreased following laparoscopic surgery. Mortality and morbidity, including colonic anastomotic leakage, were similar between groups [78]. It should be noted that quality of evidence was graded as low to very low by the Cochrane authors.

#### 4.5.2. Handsewn vs. Stapled Anastomosis

Over recent decades, many trials have focused on the influence of the surgical technique on anastomotic healing [4, 5]. Although systematic review of 1233 colorectal surgical patients in 9 RCTs comparing stapled to handsewn anastomosis shows no clear evidence of overall superiority [4], handsewn ileocolic anastomosis did correlate with more leaks than stapled anastomosis [5]. Correlating alongside the increased leak rate, ileal pouch anal anastomoses are also found to have increased learning curves when one compares handsewn versus stapled anastomotic approaches—this increased provider familiarity/technical ease of stapled ileal-anal anastomosis over handsewn has been postulated to be a confounder towards the higher handsewn leak rates [79]. Similarly, many colorectal surgeons report stapled anastomosis advantages of lower complication rates and shorter operative times [80]. Furthermore, some speculate that a higher number of leaks in handsewn colorectal anastomosis may result in a higher incidence of tumor recurrence and cancer-specific mortality [7]. Therefore, in daily practice, many surgeons base their decision of performing stapled or handsewn anastomosis on their personal preference and experience [80].

#### 4.5.3. Single vs. Double Layer Sutured Anastomosis

With varying methods to restore continuity in any intestinal anastomosis, surgeon preference generally guides care. In the setting of a manual anastomosis, approaches differ in technique and suture material. Regarding suturing technique, anastomoses may be single or double layer, which may be constructed in a continuous (faster and material-conserving) or an interrupted (slower and less narrowing) manner, which can be performed inverted or adapted or everted and may include either a portion or the entirety of the intestinal wall. For inverting stiches, the mucosa remains in the lumen, whereas everting suture protrudes the mucosa on the outside [81]. Regarding suturing material, characteristics can differ by absorbability (fast to slow absorbable and nonabsorbable) and filamentary structure (monofilament consisting of polydioxanone and multifilament made of silk, polycolic acid, polypropylene, or polyglactin). Cochrane systematic review of 7 RCTs including a total of 842 patients comparing single and double layer handsewn colorectal anastomoses revealed no difference regarding anastomotic leakage rate and mortality between the techniques. However, construction of single layer anastomosis was significantly shorter and consequently more cost-efficient [82].

#### 4.5.4. Omentoplasty

The greater omentum is a free hanging apron consisting of highly vascularized fat tissue [83]. Its beneficial characteristics have been described as early as the Napoleonic Wars [84]. Its use to cover abdominal wounds induced adhesions and thereby contained infection and prevented fatal peritonitis [84]. Therefore, a technique called “omentoplasty” was developed, using the greater omentum to cover the anastomosis [85]. Use of omentoplasty in intestinal anastomoses since the 1960s has been controversial, with proponents claiming anastomotic protection and opponents reporting disruption from adhesions and necrosis [83, 85–87]. There is currently no published Cochrane systematic review addressing the effect of omentoplasty following colorectal anastomosis, despite a Cochrane Protocol that has been published in 2008 [88]. Non-Cochrane meta-analysis of 3 RCTs including 943 patients found a reduced clinical anastomotic leakage rate in the omentoplasty group. Notably, the radiological anastomotic leakage, death, and reoperation rate were not different among groups. Given the sparse sample size, no final conclusion regarding routine use of omentoplasty was made [89].

#### 4.5.5. Intraperitoneal Agents for Preventing Adhesions

Intra-abdominal adhesions describe abnormal connections between peritoneal surfaces and usually occur following abdominal surgery in up to 95% of laparotomy patients [90]. They account for about two-thirds of all small bowel obstructions [91]. Treating surgeons are confronted with difficult access, distorted anatomy, prolonged operative time, and higher likelihood of conversion to open procedure [92]. Several different interventions aimed at preventing adhesion formation following abdominal surgery have been tested utilizing different fluid and solid phase agents [93]. These intraperitoneally applied agents should act as barriers between peritoneal surfaces. Cochrane review of 4 RCTs including 2164 patients found that intraperitoneal application of hyaluronic acid/carboxymethyl cellulose membrane reduced the incidence, severity, and extent of adhesions, but not the incidence of bowel obstructions or need for reoperation. The rate of anastomotic leakage was not affected by intraperitoneal application of these agents [93].

#### 4.5.6. Prophylactic Anastomotic Drainage

Prophylactic drains have been described since Hippocrates [94]. Their use in colonic resection was promoted in the late 19^th^ century by Sims and Billroth [95], who argued that drains served both therapeutic and diagnostic purposes by draining fluids and preventing superinfection and subsequent abscess formation [95]. Furthermore, bleeding and infectious complications may be identified earlier through sanguineous or feculent/purulent drain output [95, 96]. However, evacuation of fluids from the abdominal cavity has been shown to increase fluid (i.e., ascites) production [97], allow for outside introduction of pathogens, cause foreign material reactions (with associated peritonitis), and even cause mechanical erosion of the colonic anastomosis [98, 99]. Berliner et al. further experimentally reported that the drains prohibit the omentum from placing itself around the colonic anastomosis and thus induce anastomotic leakage [98]. Smith et al. observed the best healing of colonic anastomoses occurring when no drain was placed and that while latex drains inhibited local healing and PVC, Silastic and Teflon drains achieved intermediate results [100]. In practice, some surgeons still use drains according to their own preference. Cochrane systematic review of 2 RCTs including 809 patients revealed no difference in the colorectal anastomotic leakage rate following prophylactic drain placement [101]. Thus, in elective colon surgery, evidence does not support routine use of prophylactic drains.

#### 4.5.7. Diverting Ostomy

Diverting ostomy describes an artificial opening of the intestine to the skin. Historical reports of its use go back to the 18^th^ century, where stomas were used to relieve intestinal obstruction. Contemporary construction of an ostomy is often temporary with an intent to protect a downstream anastomosis by keeping the area clean from stool passage [102, 103]. The goal of creating a diverting ostomy is hence to reduce rates of clinically apparent colorectal anastomotic leakage and to decrease severe complications and reoperation rates [103–106]. However, diverting ostomies are associated with considerable morbidity, patient inconvenience, and hospital cost [107]. Moreover, described complications include stoma prolapse [108], stoma retraction [109, 110], intestinal adhesions [108], stenosis [107, 109], necrosis of bowel at the ostomy site [109], irritation of the surrounding skin [109–111], parastomal hernia [111], parastomal fistula [109, 110], and wound infections following stoma closure [95]. Furthermore, difficulties with stoma care may lead to isolation of patients and impairment of quality of life [109, 112]. For these reasons, many have suggested that diverting ostomy should only be performed in high risk circumstances [113], such as in total mesorectal excision and low anastomosis [114], impaired general state of health after neoadjuvant chemoradiation, steroid therapy, or underlying disease [105], and intraoperative difficulties or longer operative time [105]. To better assess, Cochrane systematic review of 6 RCTs with 648 patients undergoing low anterior resection and total mesorectal excision for rectal neoplasia revealed superiority of diverting ostomy in terms of clinically apparent leakage and reoperation rates. Regarding overall mortality, however, no difference was shown between groups with diverting ostomy and those without. These data were limited by low sample sizes, lack of independent outcome evaluators, inadequate allocation concealment, and poor methodological quality; thus, results must be interpreted with caution [115].

#### 4.5.8. Type of Ostomy

Further controversy exists regarding the most optimal type of ostomy, either an ileostomy or colostomy. It has been argued that colostomies should be associated with higher infection rates than ileostomies because bacterial counts in the small intestine are less than 1% of fecal bacterial counts, while the output from a colostomy more closely approximates normal feces [116]. Survey assessments report colostomies impair quality of life more than ileostomies due to more extensive odor, negative influence on appetite, and hygiene problems [111, 112]. In contrast, ileostomies have been shown to lose more fluid and electrolytes than colostomies [109]. Cochrane systematic review of 4 RCTs with a total of 250 patients comparing the ileostomy and colostomy for temporary decompression of left-sided colorectal anastomosis revealed no difference in anastomotic leakage, reoperation rate, wound infection, and mortality. Although stoma prolapse was more frequent in the colostomy group, according to the authors, this minor complication did not provide enough evidence to recommend one ostomy type over the other [102].

### 4.6. Postoperative Risk Factors

#### 4.6.1. Prophylactic Nasogastric Decompression

Despite early descriptions of gastric tubes during the late 18^th^ century (127), postoperative gastric tube decompression was not applied to the general practice until the 20^th^ century. Since the revisited description by Levin in 1921 and with subsequent promotion by Waldensteen in 1933, nasogastric decompression has become widely used following major gastrointestinal surgery. Early interventionalists hoped to prophylactically evacuate stomach contents to achieve a relevant reduction of emesis and gastric distention [117, 118]. Alternative theoretical advantages such as decreased pulmonary aspirations [119], decreased incisional hernias, earlier bowel recovery [120], reduced anastomotic leakage, reduced overall complication rates, and reduced mortality [121] have similarly been refuted [117]. Some studies have gone further to point out that routine nasogastric decompression is uncomfortable, unnecessary, and possibly even harmful [122]. Nevertheless, its use remains ubiquitous in practice. An updated meta-analysis of 16 RCTs with a total of 2504 patients did not provide enough evidence to justify routine postoperative nasogastric decompression [123] Although, decompression reduced episodes of emesis, routine tube insertion was also associated with notable inconvenience and morbidity, including delayed bowel recovery and increased pulmonary complications [123]. No difference was found after nasogastric decompression regarding colonic anastomotic leakage. Overall, evidence does not support routine use of prophylactic postoperative nasogastric decompression, which should only be advisable in certain clinical situations [123].

#### 4.6.2. Early Enteral Nutrition

“Nil by mouth” is a concept that was brought to surgery by the 19^th^ century. It traditionally consists of preoperative fasting the night before surgery and continues until bowel function returns. Theoretically, this practice improves anastomotic healing by minimizing stool passage [124] and reduces the risk of pulmonary aspiration and pneumonia perioperatively while anesthesia suppresses protective reflexes [63, 125]. However, clinical trials have shown that healthy individuals can endure clear fluids until two hours before operation without increased risk [126]. Similarly, early nutritional intake has been found to actually be well tolerated [127] and instead allows faster wound healing and more resistant anastomoses. This effect is thought to be due to increased availability of anabolic components, especially proteins [128, 129]. Moreover, studies have shown that early enteral feeds are associated with greater retained immunocompetence, reduced rates of infectious and septic complications [130], faster bowel recovery, better maintenance of muscle function, and shorter hospital stay [126]. A Cochrane meta-analysis update of 13 RCTs with 1232 patients comparing postoperative oral intake within 24 hours against traditional postoperative “nil by mouth” showed early feed superiority regarding length of hospital stay with complications and mortality similar between groups [131]. Accordingly, early enteral feeding did not affect the lower gastrointestinal anastomotic leakage rate.

#### 4.6.3. Epidural Anesthesia

Increased interest in enhanced recovery after surgery (ERAS) has brought increased use of epidural analgesia (EA). EA use, consisting of intrathecal application of local anesthetic agents and opioids, is associated with decreased use of systemic opioids (with consequently decreased influence on intestinal dysmotility) [132, 133]. EA use in gastrointestinal surgery is further associated with lower pain scores [133, 134], earlier return of bowel function [133–135], and shorter hospital stay [136]. Overall side effects of EA are rare [133] and include hypotension due to sympatholytic effects leading to peripheral vasodilatation [133, 137] and urinary retention [133]. Major complications of epidural abscess, persistent neural lesions (0.008%), and bleeding remain unusual [138, 139]. Cochrane review of 17 RCTs including 848 patients showed that EA use led to earlier return of gastrointestinal transit and reduced postoperative pain. Incidence of colorectal anastomotic leakage was not affected by EA [140].

## 5. Conclusion

In summary, colorectal anastomotic leakage, while often significant, can be broken down into nonalterable preexistent risks for the patient, as well as alterable factors. As further study and improved evidence are gathered regarding mutable risk factors, perioperative colorectal patient care can continue to be best optimized. Among risk factors for colorectal anastomotic leakage, utilizing high-volume operative surgeons, a stapled technique for ileocolic anastomosis, and a diverting ostomy (either ileostomy or colostomy) in surgical resection for rectal carcinoma are each shown to decrease the incidence of anastomotic failure.

## Figures and Tables

**Table 1:** Cochrane systematic reviews addressing the incidence of anastomotic leakage in colorectal surgery.

Significant/pre-, post-, postop factor	Year	Putative risk factor	Systematic review	Outcome	Leak = primary outcome?	Included studies	Participants	Anastomotic leakage (treatment group)	Anastomotic leakage (control group)	Odds ratio (CI; *P* value)	Location of anastomosis
Yes	2011	Stapled (treatment) versus handsewn (control) methods for ileocolic anastomoses [5]	Treatment = stapled	Anastomotic leak	Yes	7	1125	2.5% (11/441)	6.1% (42/684)	0.48 (CI 0.24-0.95; *P* 0.03)	Ileocolic
			Control = handsewn								

Yes	2010	Covering ostomy in anterior resection for rectal carcinoma [115]	Treatment = covering ostomy	Anastomotic leak	Yes	6	648	6.3% (21/332)	19.6% (62/316)	0.28 (CI 0.16-0.47; *P* < 0.0001)	Rectal
			Control = no covering ostomy								

Yes	2012	Impact of surgeon's operative volume on outcome after colorectal cancer surgery [33]	Treatment = high volume	Anastomotic leak	No	4	5128	4.3% (112/2576)	6.3% (162/2552)	0.67 (CI 0.49-0.92; *P* 0.012)	Colorectal
			Control = low volume								

No	2012	Impact of hospital volume on outcome after colorectal cancer surgery [33]	Treatment = high volume	Anastomotic leak	No	8	9530	6.5% (355/5435)	4.3% (176/4095)	1.18 (CI 0.87-1.58; *P* 0.29)	Colorectal
			Control = low volume								

No	2012	Impact of surgeon's specialization on outcome after colorectal cancer surgery [33]	Treatment = specialist	Anastomotic leak	No	4	9173	3.5% (195/5631)	3.8% (134/3542)	0.87 (CI 0.49-1.55; *P* 0.64)	Colorectal
			Control = no specialist								

No	2011	Mechanical bowel preparation for elective colorectal surgery [41]	Treatment = bowel prep	Anastomotic leak	Yes	13	4633	4.4% (101/2275)	4.6% (103/2258)	0.99 (CI 0.74-1.31; *P* 0.05)	Colon and rectum
			Control = no bowel prep								

No	2011	Mechanical bowel preparation for elective colorectal surgery [41]	Treatment = bowel prep	Anastomotic leak	Yes	5	1210	4.4% (27/601)	3.4% (21/609)	1.32 (CI 0.74-2.36; *P* 0.34)	Colon and rectum
			Control = rectal enema								

No	2013	Preoperative chemoradiation versus radiation alone for stage II and III resectable rectal cancer [43]	Treatment = chemoradiation	Anastomotic leak	No	4	1151	5.3% (31/588)	4.8% (27/563)	1.1 (CI 0.62-1.84; *P* 0.81)	Rectum
			Control = radiation alone								

n.a.	2004	Curative surgery for obstruction from primary left colorectal carcinoma: primary or staged resection? [18]	Treatment = primary resection	Anastomotic leak	n.a.	0	0	n.a.	n.a.	n.a.	Left colorectal
			Control = staged resection								

No	2014	Laparoscopic versus open total mesorectal excision for rectal cancer [77]	Treatment = laparoscopic	Anastomotic leak	No	10	2505	7.7% (108/1410)	6.3% (69/1095)	1.01 (CI 0.73-1.4; *P* 0.94)	Rectum
			Control = open								

No	2017	Laparoscopic versus open resection for sigmoid diverticulitis [78]	Treatment = laparoscopic	Reoperation for anastomotic leak	No	3	349	3.9% (7/180)	5.3% (9/169)	0.72 (CI 0.29-1.95; *P* 0.55)	Rectosigmoid (diverticulitis)
			Control = open								
			Control = handsewn								

No	2017	Laparoscopic versus open resection for sigmoid diverticulitis [78]	Treatment = laparoscopic	Reoperation for anastomotic leak	No	3	349	3.9% (7/180)	5.3% (9/169)	0.72 (CI 0.29-1.95; *P* 0.55)	Rectosigmoid (diverticulitis)

No	2012	Stapled versus handsewn methods for colorectal anastomosis surgery [4]	Treatment = stapled	Anastomotic leak	Yes	9	1233	13% (81/622)	13.4% (82/611)	0.97 (CI 0.7-1.35; *P* 0.84)	Colorectal



n.a.	2008	Omentoplasty for the prevention of anastomotic leakage after colonic or rectal resection [88]	Treatment = omentoplasty	n.a.	n.a.	n.a.	n.a.	n.a.	n.a.	n.a.	n.a.
			Control = no omentoplasty								

No	2012	Single (treatment) layer versus double (control) layer suture anastomosis of the gastrointestinal tract [82]	Treatment = single layer	Anastomotic leak	Yes	7	842	6.1% (25/408)	8.5% (37/434)	0.76 (CI 0.44-1.32; *P* 0.33)	Whole GI tract
			Control = double layer								

No	2009	Intraperitoneal prophylactic agents for preventing adhesions and adhesive intestinal obstruction after nongynaecological abdominal surgery [93]	Treatment = prophylactic agents	Anastomotic leak	No	5	2164	4.0% (43/1066)	2.4% (26/1098)	1.61 (CI 0.69-3.71; *P* 0.27)	Whole GI tract
			Control = no prophylactic agents								

No	2004	Prophylactic anastomotic drainage for colorectal surgery [101]	Treatment = drainage	Anastomotic leak	Yes	2	809	1.7% (7/403)	1.2% (5/406)	1.42 (CI 0.45-4.4; *P* 0.56)	Colorectal
			Control = no drainage								

No	2007	Ileostomy or colostomy for temporary decompression of left-sided colorectal anastomosis [102]	Treatment = ileostomy	Anastomotic leak	n.a.	4	250	9% (11/127)	12% (15/123)	0.72 (CI 0.36-1.47; *P* 0.52)	Left-sided colorectal
			Control = colostomy								

No	2007	Prophylactic nasogastric decompression after abdominal surgery (subanalysis colon surgery) [123]	Treatment = nasogastric decompression	Anastomotic leak	Yes	6	1122	1.7% (10/558)	1.6% (9/564)	1.13 (CI 0.46-2.74; *P* 0.79)	Colon
			Control = no decompression								

No	2018	Early enteral nutrition within 24 h of lower gastrointestinal surgery versus later commencement of feeding for length of hospital stay and postoperative complications [131]	Treatment = early enteral nutrition	Anastomotic leak	Yes	13	1232	3.3% (20/612)	4.7% (29/620)	0.68 (CI 0.39-1.23; *P* 0.21)	Colorectal
			Control = later enteral nutrition								

No	2016	Epidural local anesthetics versus opioid-based analgesic regimens for abdominal surgery [140]	Treatment = epidural	Anastomotic leak	No	17	848	3.6% (16/433)	5.3% (22/415)	0.69 (CI 0.35-1.32; *P* 0.26)	Whole GI tract
			Control = opioids								

## References

[1] Senn N. (1893). Enterorrhaphy; its history, technique and present status. *JAMA*.

[2] Dietz U., Russo S. (2002). Resektions- und Rekonstruktionsverfahren in der Viszeralchirurgie: Lehrbuch und Atlas der biofragmentierbaren Anastomose. *Kaden Verlag*.

[3] Ravitch M. M., Steichen F. M. (1979). A stapling instrument for end-to-end inverting anastomoses in the gastrointestinal tract. *Annals of Surgery*.

[4] Neutzling C. B., Lustosa S. A., Proenca I. M., da Silva E. M., Matos D. (2012). Stapled versus handsewn methods for colorectal anastomosis surgery. *The Cochrane Database of Systematic Reviews*.

[5] Choy P. Y., Bissett I. P., Docherty J. G., Parry B. R., Merrie A., Fitzgerald A. (2011). Stapled versus handsewn methods for ileocolic anastomoses. *Cochrane Database of Systematic Reviews*.

[6] Brundage S. I., Jurkovich G. J., Hoyt D. B. (2001). Stapled versus sutured gastrointestinal anastomoses in the trauma patient: a multicenter trial. *The Journal of Trauma*.

[7] Docherty J. G., McGregor J. R., Akyol A. M., Murray G. D., Galloway D. J. (1995). Comparison of manually constructed and stapled anastomoses in colorectal surgery. West of Scotland and Highland Anastomosis Study Group. *Annals of Surgery*.

[8] Boccola M. A., Lin J., Rozen W. M., Ho Y. H. (2010). Reducing anastomotic leakage in oncologic colorectal surgery: an evidence-based review. *Anticancer Research*.

[9] Kube R., Mroczkowski P., Granowski D. (2010). Anastomotic leakage after colon cancer surgery: A predictor of significant morbidity and hospital mortality, and diminished tumour-free survival. *European Journal of Surgical Oncology (EJSO)*.

[10] Bruce J., Krukowski Z. H., Al-Khairy G., Russell E. M., Park K. G. (2001). Systematic review of the definition and measurement of anastomotic leak after gastrointestinal surgery. *British Journal of Surgery*.

[11] Matthiessen P., Hallbook O., Andersson M., Rutegard J., Sjodahl R. (2004). Risk factors for anastomotic leakage after anterior resection of the rectum. *Colorectal Disease*.

[12] Rahbari N. N., Weitz J., Hohenberger W. (2010). Definition and grading of anastomotic leakage following anterior resection of the rectum: a proposal by the International Study Group of Rectal Cancer. *Surgery*.

[13] Makela J. T., Kiviniemi H., Laitinen S. (2003). Risk factors for anastomotic leakage after left-sided colorectal resection with rectal anastomosis. *Diseases of the Colon and Rectum*.

[14] Lynn E. T., Chen J., Wilck E. J., El-Sabrout K., Lo C. C., Divino C. M. (2013). Radiographic findings of anastomotic leaks. *The American Surgeon*.

[15] Kornmann V. N., van Ramshorst B., Smits A. B., Bollen T. L., Boerma D. (2014). Beware of false-negative CT scan for anastomotic leakage after colonic surgery. *International Journal of Colorectal Disease*.

[16] Choi H. K., Law W. L., Ho J. W. (2006). Leakage after resection and intraperitoneal anastomosis for colorectal malignancy: analysis of risk factors. *Diseases of the Colon and Rectum*.

[17] McDermott F. D., Heeney A., Kelly M. E., Steele R. J., Carlson G. L., Winter D. C. (2015). Systematic review of preoperative, intraoperative and postoperative risk factors for colorectal anastomotic leaks. *The British Journal of Surgery*.

[18] De Salvo G. L., Gava C., Pucciarelli S., Lise M. (2004). Curative surgery for obstruction from primary left colorectal carcinoma: primary or staged resection?. *Cochrane Database of Systematic Reviews*.

[19] Penninckx F., Beirens K., Fieuws S. (2012). Risk adjusted benchmarking of clinical anastomotic leakage rate after total mesorectal excision in the context of an improvement project. *Colorectal Disease*.

[20] Rullier E., Laurent C., Garrelon J. L., Michel P., Saric J., Parneix M. (1998). Risk factors for anastomotic leakage after resection of rectal cancer. *The British Journal of Surgery*.

[21] Jonsson K., Jensen J. A., Goodson W. H. (1991). Tissue oxygenation, anemia, and perfusion in relation to wound healing in surgical patients. *Annals of Surgery*.

[22] Fielding L. P., Stewart-Brown S., Blesovsky L., Kearney G. (1980). Anastomotic integrity after operations for large-bowel cancer: a multicentre study. *British Medical Journal*.

[23] Sorensen L. T., Jorgensen T., Kirkeby L. T., Skovdal J., Vennits B., Wille-Jorgensen P. (1999). Smoking and alcohol abuse are major risk factors for anastomotic leakage in colorectal surgery. *The British Journal of Surgery*.

[24] Alves A., Panis Y., Trancart D., Regimbeau J. M., Pocard M., Valleur P. (2002). Factors associated with clinically significant anastomotic leakage after large bowel resection: multivariate analysis of 707 patients. *World Journal of Surgery*.

[25] Golub R., Golub R. W., Cantu R., Stein H. D. (1997). A multivariate analysis of factors contributing to leakage of intestinal anastomoses. *Journal of the American College of Surgeons*.

[26] Kasperk R., Philipps B., Vahrmeyer M., Willis S. (2000). Risikofaktoren der Anastomoseninsuffizienz nach sehr tiefer colorectaler und coloanaler Anastomose: Risk factors for anastomotic dehiscence after very low colorectal and coloanal anastomosis. *Der Chirurg*.

[27] Mandai R., Eguchi Y., Tanaka M., Sai Y., Nosaka S. (2001). Effects of profound hemodilution on small-intestinal wound healing in rabbits. *The Journal of Surgical Research*.

[28] Bruewer M., Utech M., Rijcken E. J. (2003). Preoperative steroid administration: effect on morbidity among patients undergoing intestinal bowel resection for Crohńs disease. *World Journal of Surgery*.

[29] Burns J. L., Mancoll J. S., Phillips L. G. (2003). Impairments to wound healing. *Clinics in Plastic Surgery*.

[30] Mann B., Kleinschmidt S., Stremmel W. (1996). Prospective study of hand-sutured anastomosis after colorectal resection. *The British Journal of Surgery*.

[31] Vuille-dit-Bille R. N., Wallace B., Fink L. (2018). Trainee factors influencing initial performance in single incision pediatric endoscopic surgery simulation training. *Clinics in Surgery*.

[32] Leu S., Staerkle R. F., Gaukel S. (2019). Impact of sleep deprivation on surgical laparoscopic performance in Novices. *Surgical Laparoscopy, Endoscopy & Percutaneous Techniques*.

[33] Archampong D., Borowski D., Wille-Jorgensen P., Iversen L. H. (2012). Workload and surgeon´s specialty for outcome after colorectal cancer surgery. *Cochrane Database of Systematic Reviews*.

[34] Turrentine F. E., Denlinger C. E., Simpson V. B. (2015). Morbidity, mortality, cost, and survival estimates of gastrointestinal anastomotic leaks. *Journal of the American College of Surgeons*.

[35] Rosenberg I. L., Graham N. G., De Dombal F. T., Goligher J. C. (1971). Preparation of the intestine in patients undergoing major large-bowel surgery, mainly for neoplasms of the colon and rectum. *The British Journal of Surgery*.

[36] Smith S. R., Connolly J. C., Gilmore O. J. (1983). The effect of faecal loading on colonic anastomotic healing. *The British Journal of Surgery*.

[37] Nichols R. L., Condon R. E. (1971). Preoperative preparation of the colon. *Surgery, Gynecology & Obstetrics*.

[38] Hughes E. S. (1972). Asepsis in large-bowel surgery. *Annals of the Royal College of Surgeons of England*.

[39] Jung B., Pahlman L., Nystrom P. O., Nilsson E. (2007). Multicentre randomized clinical trial of mechanical bowel preparation in elective colonic resection. *The British Journal of Surgery*.

[40] Bretagnol F., Panis Y., Rullier E. (2010). Rectal cancer surgery with or without bowel preparation: the French GRECCAR III multicenter single-blinded randomized trial. *Annals of Surgery*.

[41] Guenaga K. F., Matos D., Wille-Jorgensen P. (2011). Mechanical bowel preparation for elective colorectal surgery. *Cochrane Database of Systematic Reviews*.

[42] Wong R. K., Tandan V., De Silva S., Figueredo A. (2007). Pre-operative radiotherapy and curative surgery for the management of localized rectal carcinoma. *Cochrane Database of Systematic Reviews*.

[43] De Caluwe L., Van Nieuwenhove Y., Ceelen W. P. (2013). Preoperative chemoradiation versus radiation alone for stage II and III resectable rectal cancer. *Cochrane Database of Systematic Reviews*.

[44] Quirke P., Durdey P., Dixon M. F., Williams N. S. (1986). Local recurrence of rectal adenocarcinoma due to inadequate surgical resection. Histopathological study of lateral tumour spread and surgical excision. *Lancet*.

[45] Heald R. J., Moran B. J., Ryall R. D., Sexton R., MacFarlane J. K. (1998). Rectal cancer: the Basingstoke experience of total mesorectal excision, 1978-1997. *Archives of Surgery*.

[46] Colorectal Cancer Collaborative G (2001). Adjuvant radiotherapy for rectal cancer: a systematic overview of 8507 patients from 22 randomised trials. *The Lancet*.

[47] Glimelius B. (2002). Radiotherapy in rectal cancer. *British Medical Bulletin*.

[48] Minsky B. D., Cohen A. M., Enker W. E. (1997). Preoperative 5-FU, low-dose leucovorin, and radiation therapy for locally advanced and unresectable rectal cancer. *International Journal of Radiation Oncology, Biology, Physics*.

[49] Nagtegaal I. D., Marijnen C. A., Kranenbarg E. K., van de Velde C. J., van Krieken J. H. (2002). Circumferential margin involvement is still an important predictor of local recurrence in rectal carcinoma: not one millimeter but two millimeters is the limit. *The American Journal of Surgical Pathology*.

[50] Kapiteijn E., Marijnen C. A., Nagtegaal I. D. (2001). Preoperative radiotherapy combined with total mesorectal excision for resectable rectal cancer. *The New England Journal of Medicine*.

[51] Dudley R. A., Johansen K. L., Brand R., Rennie D. J., Milstein A. (2000). Selective referral to high-volume hospitals: estimating potentially avoidable deaths. *JAMA*.

[52] Chioreso C., Del Vecchio N., Schweizer M. L., Schlichting J., Gribovskaja-Rupp I., Charlton M. E. (2018). Association Between Hospital and Surgeon Volume and Rectal Cancer Surgery Outcomes in Patients With Rectal Cancer Treated Since 2000: Systematic Literature Review and Meta-analysis. *Diseases of the Colon and Rectum*.

[53] Ihse I. (2003). The volume-outcome relationship in cancer surgery: a hard sell. *Annals of Surgery*.

[54] Ohman U. (1982). Prognosis in patients with obstructing colorectal carcinoma. *American Journal of Surgery*.

[55] Serpell J. W., McDermott F. T., Katrivessis H., Hughes E. S. (1989). Obstructing carcinomas of the colon. *The British Journal of Surgery*.

[56] Deans G. T., Krukowski Z. H., Irwin S. T. (1994). Malignant obstruction of the left colon. *The British Journal of Surgery*.

[57] Phillips R. K., Hittinger R., Fry J. S., Fielding L. P. (1985). Malignant large bowel obstruction. *The British Journal of Surgery*.

[58] Fielding L. P., Wells B. W. (1974). Survival after primary and after staged resection for large bowel obstruction caused by cancer. *The British Journal of Surgery*.

[59] Runkel N. S., Schlag P., Schwarz V., Herfarth C. (1991). Outcome after emergency surgery for cancer of the large intestine. *The British Journal of Surgery*.

[60] Zhao X. D., Cai B. B., Cao R. S., Shi R. H. (2013). Palliative treatment for incurable malignant colorectal obstructions: a meta-analysis. *World Journal of Gastroenterology*.

[61] Tilney H. S., Lovegrove R. E., Purkayastha S. (2007). Comparison of colonic stenting and open surgery for malignant large bowel obstruction. *Surgical Endoscopy*.

[62] Sebastian S., Johnston S., Geoghegan T., Torreggiani W., Buckley M. (2004). Pooled analysis of the efficacy and safety of self-expanding metal stenting in malignant colorectal obstruction. *The American Journal of Gastroenterology*.

[63] Wholey M. H., Levine E. A., Ferral H., Castaneda-Zuniga W. (1998). Initial clinical experience with colonic stent placement. *American Journal of Surgery*.

[64] de Gregorio M. A., Mainar A., Tejero E. (1998). Acute colorectal obstruction: stent placement for palliative treatment--results of a multicenter study. *Radiology*.

[65] Cole B., Parker O.,. S. (2000). Endoluminal stenting for relief of colonic obstruction is safe and effective. *Colorectal Disease*.

[66] Mainar A., De Gregorio Ariza M. A., Tejero E. (1999). Acute colorectal obstruction: treatment with self-expandable metallic stents before scheduled surgery--results of a multicenter study. *Radiology*.

[67] Ribeiro I. B., Bernardo W. M., Martins B. D. C. (2018). Colonic stent versus emergency surgery as treatment of malignant colonic obstruction in the palliative setting: a systematic review and meta-analysis. *Endoscopy International Open*.

[68] Bertagnolli M. M., Mahmoud N. N., Daly J. M. (1997). Surgical aspects of colorectal carcinoma. *Hematology/Oncology Clinics of North America*.

[69] Holzman M. D., Eubanks S. (1997). Laparoscopic Colectomy: Prospects and Problems. *Gastrointestinal Endoscopy Clinics of North America*.

[70] Jacobs M., Verdeja J. C., Goldstein H. S. (1991). Minimally invasive colon resection (laparoscopic colectomy). *Surgical Laparoscopy & Endoscopy*.

[71] Lacy A. M., Garcia-Valdecasas J. C., Delgado S. (2002). Laparoscopy-assisted colectomy versus open colectomy for treatment of non-metastatic colon cancer: a randomised trial. *Lancet*.

[72] Obrist N. M., Tschuor C., Breitenstein S., Vuille-Dit-Bille R. N., Soll C. (2019). Appendectomy in Switzerland: how is it done?. *Updates in Surgery*.

[73] Vuille-dit-Bille R. N., Soll C., Mazel P., Staerkle R. F., Breitenstein S. (2019). Appendiceal stump closure with polymeric clips is a reliable alternative to endostaplers. *Journal of International Medical Research*.

[74] Wenk K., Humoud I., Fink L. (2017). Open versus laparoscopic pyloromyotomy for pyloric stenosis. *Cochrane Database of Systematic Reviews*.

[75] Dewberry L. C., Vuille-Dit-Bille R. N., Kulungowski A. M., Somme S. (2018). A single surgeon laparoscopic duodenoduodenostomy case series for congenital duodenal obstruction in an academic setting. *Journal of Laparoendoscopic & Advanced Surgical Techniques*.

[76] Tekkis P. P., Senagore A. J., Delaney C. P., Fazio V. W. (2005). Evaluation of the learning curve in laparoscopic colorectal surgery: comparison of right-sided and left-sided resections. *Annals of Surgery*.

[77] Vennix S., Pelzers L., Bouvy N. (2014). Laparoscopic versus open total mesorectal excision for rectal cancer. *Cochrane Database of Systematic Reviews*.

[78] Abraha I., Binda G. A., Montedori A., Arezzo A., Cirocchi R. (2017). Laparoscopic versus open resection for sigmoid diverticulitis. *Cochrane Database of Systematic Reviews*.

[79] Tekkis P. P., Fazio V. W., Lavery I. C. (2005). Evaluation of the learning curve in ileal pouch-anal anastomosis surgery. *Annals of Surgery*.

[80] Korolija D. (2008). The current evidence on stapled versus hand-sewn anastomoses in the digestive tract. *Minimally Invasive Therapy & Allied Technologies*.

[81] Goligher J. C., Morris C., McAdam W. A., De Dombal F. T., Johnston D. (1970). A controlled trial of inverting versus everting intestinal suture in clinical large-bowel surgery. *The British Journal of Surgery*.

[82] Sajid M. S., Siddiqui M. R., Baig M. K. (2012). Single layer versus double layer suture anastomosis of the gastrointestinal tract. *Cochrane Database of Systematic Reviews*.

[83] HS G. (1990). The Omentum. Research and Clinical Applications. *Springer, New York Berlin Heidelberg*.

[84] WH L.-M. D. (1983). The Greater Omentum. Anatomy, Physiology, Pathology, Surgery, with an Historical Survey. *Springer, Berlin Heidelberg New York*.

[85] Smith S. R., Swift I., Gompertz H., Baker W. N. (1988). Abdominoperineal and anterior resection of the rectum with retrocolic omentoplasty and no drainage. *The British Journal of Surgery*.

[86] Carter D. C., Jenkins D. H., Whitfield H. N. (1972). Omental reinforcement of intestinal anastomoses. An experimental study in the rabbit. *The British Journal of Surgery*.

[87] Jakowatz J. G., Porudominsky D., Riihimaki D. U. (1985). Complications of pelvic exenteration. *Archives of Surgery*.

[88] Herrle F., Schattenberg T. (2008). Omentoplasty for the prevention of anastomotic leakage after colonic or rectal resection. *Cochrane Database of Systematic Reviews*.

[89] Hao X. Y., Yang K. H., Guo T. K., Ma B., Tian J. H., Li H. L. (2008). Omentoplasty in the prevention of anastomotic leakage after colorectal resection: a meta-analysis. *International Journal of Colorectal Disease*.

[90] Ellis H. (1997). The clinical significance of adhesions: focus on intestinal obstruction. *The European Journal of Surgery. Supplement*.

[91] Menzies D. (1992). Peritoneal adhesions. Incidence, cause, and prevention. *Surg Annu*.

[92] Coleman M. G., McLain A. D., Moran B. J. (2000). Impact of previous surgery on time taken for incision and division of adhesions during laparotomy. *Diseases of the Colon and Rectum*.

[93] Kumar S., Wong P. F., Leaper D. J. (2009). Intra-peritoneal prophylactic agents for preventing adhesions and adhesive intestinal obstruction after non-gynaecological abdominal surgery. *Cochrane Database of Systematic Reviews*.

[94] Robinson J. O. (1986). Surgical drainage: an historical perspective. *The British Journal of Surgery*.

[95] Averbach A. M., Sugarbaker P. H. (1995). The use of drains in elective surgery for colorectal cancer: always, never or selectively?. *Tumori*.

[96] Lennox M. S. (1984). Prophylactic drainage of colonic anastomoses. *The British Journal of Surgery*.

[97] Elboim C. M., Goldman L., Hann L., Palestrant A. M., Silen W. (1983). Significance of post-cholecystectomy subhepatic fluid collections. *Annals of Surgery*.

[98] Berliner S. D., Burson L. C., Lear P. E. (1964). Use and abuse of intraperitoneal drains in colon surgery. *Archives of Surgery*.

[99] Shear L., Swartz C., Shinaberger J. A., Barry K. G. (1965). Kinetics of peritoneal fluid absorption in adult man. *The New England Journal of Medicine*.

[100] Smith S. R., Connolly J. C., Crane P. W., Gilmore O. J. (1982). The effect of surgical drainage materials on colonic healing. *The British Journal of Surgery*.

[101] Jesus E. C., Karliczek A., Matos D., Castro A. A., Atallah A. N. (2004). Prophylactic anastomotic drainage for colorectal surgery. *Cochrane Database of Systematic Reviews*.

[102] Guenaga K. F., Lustosa S. A., Saad S. S., Saconato H., Matos D. (2008). Ileostomy or colostomy for temporary decompression of colorectal anastomosis. Systematic review and meta-analysis. *Acta Cirúrgica Brasileira*.

[103] Vuille-dit-Bille R. N., Berger C., Meuli M., Grotzer M. A. (2016). Colostomy for perianal sepsis with ecthyma gangrenosum in immunocompromised children. *Journal of Pediatric Hematology/Oncology*.

[104] Matthiessen P., Hallbook O., Rutegard J., Simert G., Sjodahl R. (2007). Defunctioning stoma reduces symptomatic anastomotic leakage after low anterior resection of the rectum for cancer: a randomized multicenter trial. *Annals of Surgery*.

[105] Marusch F., Koch A., Schmidt U. (2002). Value of a protective stoma in low anterior resections for rectal cancer. *Diseases of the Colon and Rectum*.

[106] Pakkastie T. E., Ovaska J. T., Pekkala E. S., Luukkonen P. E., Jarvinen H. J. (1997). A randomised study of colostomies in low colorectal anastomoses. *The European Journal of Surgery*.

[107] Graffner H., Fredlund P., Olsson S. A., Oscarson J., Petersson B. G. (1983). Protective colostomy in low anterior resection of the rectum using the EEA stapling instrument. A randomized study. *Diseases of the Colon and Rectum*.

[108] Edwards D. P., Leppington-Clarke A., Sexton R., Heald R. J., Moran B. J. (2001). Stoma-related complications are more frequent after transverse colostomy than loop ileostomy: a prospective randomized clinical trial. *The British Journal of Surgery*.

[109] Gooszen A. W., Geelkerken R. H., Hermans J., Lagaay M. B., Gooszen H. G. (1998). Temporary decompression after colorectal surgery: randomized comparison of loop ileostomy and loop colostomy. *The British Journal of Surgery*.

[110] Khoury G. A., Lewis M. C., Meleagros L., Lewis A. A. (1987). Colostomy or ileostomy after colorectal anastomosis?: a randomised trial. *Annals of the Royal College of Surgeons of England*.

[111] Williams N. S., Nasmyth D. G., Jones D., Smith A. H. (1986). De-functioning stomas: a prospective controlled trial comparing loop ileostomy with loop transverse colostomy. *The British Journal of Surgery*.

[112] Silva M. A., Ratnayake G., Deen K. I. (2003). Quality of life of stoma patients: temporary ileostomy versus colostomy. *World Journal of Surgery*.

[113] Wong N. Y., Eu K. W. (2005). A defunctioning ileostomy does not prevent clinical anastomotic leak after a low anterior resection: a prospective, comparative study. *Diseases of the Colon and Rectum*.

[114] Laxamana A., Solomon M. J., Cohen Z., Feinberg S. M., Stern H. S., McLeod R. S. (1995). Long-term results of anterior resection using the double-stapling technique. *Diseases of the Colon and Rectum*.

[115] Montedori A., Cirocchi R., Farinella E., Sciannameo F., Abraha I. (2010). Covering ileo- or colostomy in anterior resection for rectal carcinoma. *Cochrane Database of Systematic Reviews*.

[116] Gorbach S. L., Tabaqchali S. (1969). Bacteria, bile, and the small bowel. *Gut*.

[117] Cheatham M. L., Chapman W. C., Key S. P., Sawyers J. L. (1995). A meta-analysis of selective versus routine nasogastric decompression after elective laparotomy. *Annals of Surgery*.

[118] Montgomery R. C., Bar-Natan M. F., Thomas S. E., Cheadle W. G. (1996). Postoperative nasogastric decompression: a prospective randomized trial. *Southern Medical Journal*.

[119] Miller D. F., Mason J. R., McArthur J., Gordon I. (1972). A randomized prospective trial comparing three established methods of gastric decompression after vagotomy. *The British Journal of Surgery*.

[120] Cunningham J., Temple W. J., Langevin J. M., Kortbeek J. (1992). A prospective randomized trial of routine postoperative nasogastric decompression in patients with bowel anastomosis. *Canadian Journal of Surgery*.

[121] Jamieson W. G., DeRose G., Harris K. A. (1992). Routine nasogastric decompression after abdominal surgery?. *Canadian Journal of Surgery*.

[122] Argov S., Goldstein I., Barzilai A. (1980). Is routine use of the nasogastric tube justified in upper abdominal surgery?. *American Journal of Surgery*.

[123] Nelson R., Edwards S., Tse B. (2007). Prophylactic nasogastric decompression after abdominal surgery. *Cochrane Database of Systematic Reviews*.

[124] Fukuzawa J., Terashima H., Ohkohchi N. (2007). Early postoperative oral feeding accelerates upper gastrointestinal anastomotic healing in the rat model. *World Journal of Surgery*.

[125] HC B. H. (1923). *Practical Anaesthetics*.

[126] Maltby J. R. (2006). Fasting from midnight--the history behind the dogma. *Best Practice & Research. Clinical Anaesthesiology*.

[127] McCarter M. D., Gomez M. E., Daly J. M. (1997). Early postoperative enteral feeding following major upper gastrointestinal surgery. *Journal of Gastrointestinal Surgery*.

[128] Wilmore D. W. (2000). Metabolic response to severe surgical illness: overview. *World Journal of Surgery*.

[129] Moss G. (1981). Maintenance of gastrointestinal function after bowel surgery and immediate enteral full nutrition. II. Clinical experience, with objective demonstration of intestinal absorption and motility. *JPEN Journal of Parenteral and Enteral Nutrition*.

[130] Moore F. A., Moore E. E., Jones T. N., McCroskey B. L., Peterson V. M. (1989). TEN versus TPN following major abdominal trauma--reduced septic morbidity. *The Journal of Trauma*.

[131] Herbert G., Perry R., Andersen H. K. (2018). Early enteral nutrition within 24 hours of lower gastrointestinal surgery versus later commencement for length of hospital stay and postoperative complications. *Cochrane Database of Systematic Reviews*.

[132] Wongyingsinn M., Baldini G., Charlebois P., Liberman S., Stein B., Carli F. (2011). Intravenous lidocaine versus thoracic epidural analgesia: a randomized controlled trial in patients undergoing laparoscopic colorectal surgery using an enhanced recovery program. *Regional Anesthesia and Pain Medicine*.

[133] Zingg U., Miskovic D., Hamel C. T., Erni L., Oertli D., Metzger U. (2009). Influence of thoracic epidural analgesia on postoperative pain relief and ileus after laparoscopic colorectal resection : benefit with epidural analgesia. *Surgical Endoscopy*.

[134] Taqi A., Hong X., Mistraletti G., Stein B., Charlebois P., Carli F. (2007). Thoracic epidural analgesia facilitates the restoration of bowel function and dietary intake in patients undergoing laparoscopic colon resection using a traditional, nonaccelerated, perioperative care program. *Surgical Endoscopy*.

[135] Kuo C. P., Jao S. W., Chen K. M. (2006). Comparison of the effects of thoracic epidural analgesia and i.v. infusion with lidocaine on cytokine response, postoperative pain and bowel function in patients undergoing colonic surgery. *British Journal of Anaesthesia*.

[136] Senagore A. J., Whalley D., Delaney C. P., Mekhail N., Duepree H. J., Fazio V. W. (2001). Epidural anesthesia-analgesia shortens length of stay after laparoscopic segmental colectomy for benign pathology. *Surgery*.

[137] Holte K., Foss N. B., Svensen C., Lund C., Madsen J. L., Kehlet H. (2004). Epidural anesthesia, hypotension, and changes in intravascular volume. *Anesthesiology*.

[138] Phillips O. C., Ebner H., Nelson A. T., Black M. H. (1969). Neurologic complications following spinal anesthesia with lidocaine: a prospective review of 10,440 cases. *Anesthesiology*.

[139] Baker A. S., Ojemann R. G., Swartz M. N., Richardson E. P. (1975). Spinal epidural abscess. *The New England Journal of Medicine*.

[140] Guay J., Nishimori M., Kopp S. (2016). Epidural local anaesthetics versus opioid-based analgesic regimens for postoperative gastrointestinal paralysis, vomiting and pain after abdominal surgery. *Cochrane Database of Systematic Reviews*.

